# 

**Published:** 1894-04

**Authors:** 


### NOTICES.

### The San Francisco Dental Association.

The San Francisco Dental Association holds its meetings at Union Square Hall, Post Street, on the second Monday evening of each month.

All members of the profession, and especially visitors from abroad and from the interior, are cordially invited to attend these meetings and to contribute to their success.

The officers for the present year are: Dr. C. L. Goddard, President; Dr. Frank C. Pague, Secretary; and Dr. G. N. Van Orden, Corresponding Secretary.

The annual meeting and election of officers is held in October.

### Joint Meeting of the Iowa and Nebraska State Dental Societies.

The annual meeting of the Iowa and Nebraska State Dental Societies will be a joint meeting held at Council Bluffs, Iowa, and Omaha, Nebraska, May 1st to 4th, inclusive. An interesting programme will be presented. All are cordially invited to attend.

W. C. DAVIS, *Sec. Nebraska Society,*
LINCOLN, NEBRASKA.
F. T. BREENE, *Sec. Iowa Society,*
IOWA CITY, IOWA.

### Northern Ohio Dental Association.

The thirty-fifth annual meeting of the Northern Ohio Dental Association will be held at the Beebe House, Put-in-Bay Island, on Tuesday, June 19, 1894, at 2 o'clock P. M., (instead of the second Tuesday in May as formerly announced,) continuing in session three days.

A very interesting programme has been arranged for this occasion, including papers and discussion upon the following subjects: Immediate Root Filling; Systemic Disturbances and their Relation to Dental Diseases; Correcting Irregularities of the Dental Arch; Local Anæsthetics; Porcelain Work; Things New in Dentistry; etc.

Special arrangements have been made with the proprietors of the Beebe House, whereby reduced rates have been secured, also the use of rooms for meetings and clinics.

A steamer will leave Cleveland at 8 A. M. daily and arrive at the Island at 12 M. Returning will leave the Island at 4 P. M. and arrive at Cleveland at 8:30 P. M. At Sandusky boats leave and return at all hours.

A cordial invitation is extended to all members of the profession to be present.

### THE DENTAL REGISTER,

A MONTHLY MAGAZINE DEVOTED TO THE INTERESTS OF THE

**DENTAL PROFESSION.**

*Terms Reduced to $2.00 Per Year. Single Copies, 25 Cents.*

**EDITED BY PROF. J. TAFT, D.D.S.**

—AND—

**Published by SAM'L A. CROCKER & CO., Ohio Dental and Surgical Depot.**

The volume commences in January and closes in December.

The REGISTER being issued monthly is always fresh, therefore Indispensable to the practitioner who desires to keep posted up with the new inventions and improvements which are daily developed. Neither expense nor effort will be spared to give value and variety to its contents Articles will be illustrated with well executed engravings whenever they will add to the interest or assist to explanation.

Its extensive circulation and frequency of issue make it one of the BEST DENTAL ADVERTISING MEDIUMS in the United States.

All subscriptions must begin with January or July.

Local or special notices, five lines or less, will be inserted for $3 each insertion; over five lines, 50 cts. per line.

All advertising bills payable in advance, or at furthest, after the issue of the first number containing the advertisement. All transient advertisements to be paid for in advance.

☞CONTRIBUTIONS SOLICITED.

Exclusively Editorial Communications should be addressed to the Editor.

The latest number of the REGISTER may be ordered with or without other good at any time, and all letters should be addressed to

